# Investigating resting brain perfusion abnormalities and disease target-engagement by intranasal oxytocin in women with bulimia nervosa and binge-eating disorder and healthy controls

**DOI:** 10.1038/s41398-020-00871-w

**Published:** 2020-06-08

**Authors:** Daniel Martins, Monica Leslie, Sarah Rodan, Fernando Zelaya, Janet Treasure, Yannis Paloyelis

**Affiliations:** 1 Department of Neuroimaging, Institute of Psychiatry, Psychology and Neuroscience, King’s College London, De Crespigny Park, London, SE5 8AF UK; 2 Section of Eating Disorders, Institute of Psychiatry, Psychology and Neuroscience, King’s College London, De Crespigny Park, London, SE5 8AF UK

**Keywords:** Psychiatric disorders, Biomarkers, Pharmacology, Psychiatric disorders, Biomarkers

## Abstract

Advances in the treatment of bulimia nervosa and binge-eating disorder (BN/BED) have been marred by our limited understanding of the underpinning neurobiology. Here we measured regional cerebral blood flow (rCBF) to map resting perfusion abnormalities in women with BN/BED compared with healthy controls and investigate whether intranasal oxytocin (OT), proposed as a potential treatment, can restore perfusion in disorder-related brain circuits. Twenty-four women with BN/BED and 23 healthy women participated in a randomized, double-blind, crossover, placebo-controlled study. We used arterial spin labelling MRI to measure rCBF and the effects of an acute dose of intranasal OT (40 IU) or placebo over 18–26 min post dosing, as we have previously shown robust OT-induced changes in resting rCBF in men in a similar time-window (15–36 min post dosing). We tested for effects of treatment, diagnosis and their interaction on extracted rCBF values in anatomical regions-of-interest previously implicated in BN/BED by other neuroimaging modalities, and conducted exploratory whole-brain analyses to investigate previously unidentified brain regions. We demonstrated that women with BN/BED presented increased resting rCBF in the medial prefrontal and orbitofrontal cortices, anterior cingulate gyrus, posterior insula and middle/inferior temporal gyri bilaterally. Hyperperfusion in these areas specifically correlated with eating symptoms severity in patients. Our data did not support a normalizing effect of intranasal OT on perfusion abnormalities in these patients, at least for the specific dose (40 IU) and post-dosing interval (18–26 min) examined. Our findings enhance our understanding of resting brain abnormalities in BN/BED and identify resting rCBF as a non-invasive potential biomarker for disease-related changes and treatment monitoring. They also highlight the need for a comprehensive investigation of intranasal OT pharmacodynamics in women before we can fully ascertain its therapeutic value in disorders affecting predominantly this gender, such as BN/BED.

## Introduction

Bulimia nervosa (BN) and binge-eating disorder (BED) are psychiatric disorders characterized by recurrent binge eating^1^. In BN, binge eating is accompanied by excessive compensatory behaviours, which are absent in BED^2^. As recovery rates after standard treatment remain poor in both disorders^3^, developing new effective treatments that address binge eating are needed; however, progress is impeded by our limited understanding of the neurobiology of BN/BED.

BN/BED have been conceptualized as impulsive/compulsive eating disorders^4^ with altered reward sensitivity^5^ and food-related attentional biases^6^. Consistent with this model of BN/BED, neuroimaging studies in these patients have highlighted changes in the function, structure and neurochemistry in brain areas involved in incentive processing (such as the orbitofrontal cortex (OFC), ventral tegmental area (VTA), substantia nigra (SN), nucleus accumbens and the amygdala), inhibitory control (such as the medial prefrontal cortex (PFC) and the anterior cingulate (ACG)) and habitual behaviour (such as the dorsal striatum) (see refs. ^7,8^ for detailed reviews). Most of these studies focused on morphological differences between BN/BED patients and healthy controls. Some studies investigated functional abnormalities using task-based BOLD functional magnetic resonance imaging (fMRI), allowing for inferences that are restricted to the neural circuits employed by the specific tasks. Other studies investigated alterations in spontaneous fluctuations of the BOLD signal at rest, suggesting aberrant functional connectivity in brain networks involved in salience attribution, self-referential processing and cognitive control in people with BN/BED^9–13^). However, BOLD fMRI (whether focusing on fluctuations at rest or task-evoked changes) typically yields only relative metrics and is prone to several artefacts, including motion, low-frequency physiological noise and baseline drift^14^. Three other studies, all with small sample sizes, used single-photon emission computerized tomography (SPECT) to measure regional cerebral blood flow (rCBF), which provides a proxy of brain metabolism and neural activity^15^. These studies reported rCBF increases in the temporal lobes and/or the frontal cortex of patients with BN and BED in response to anticipation or exposure to food/body shape-related stimuli^16–18^. However, SPECT is a high-cost technique that requires the injection of a radionuclide tracer, making it suboptimal as a routine screening tool in non-specialized centres or for evaluating short-term responses^19^.

Recently, arterial spin labelling (ASL) MRI has allowed the non-invasive quantification of rCBF with high reproducibility and spatial resolution^20^. ASL is a quantitative technique and has been used widely to quantify changes in brain physiology associated with various neuropsychiatric disorders^21^ (e.g., autism spectrum disorder^22^, schizophrenia^23^, depression^24^) and in response to pharmacological treatments^25,26^. Hence, ASL provides a non-invasive and widely available tool to investigate abnormalities in resting brain physiology of BN/BED and the effects of potential treatments.

One such potential treatment is intranasal oxytocin (OT)^27–29^. The implication of OT in binge eating is supported by a wide range of evidence. Candidate gene studies have found associations between polymorphisms in the OT receptor gene and binge-purge tendencies in women (rs53576/rs2254298)^30^, bulimia (rs53576)^31^ and overeating (rs2268493/rs2268494)^32^. Similarly, Prader–Willi syndrome patients present with a reduction in OT-synthesizing neurons in the paraventricular nucleus of the hypothalamus^33^, which is accompanied by compulsive overeating behaviour from early childhood^34^. However, the only two studies conducted to compare the levels of OT in the cerebrospinal fluid of patients with BN and healthy controls could not find any differences^35,36^. Pharmacological animal^37^ and human studies^38,39^ have demonstrated that OT suppresses eating, including hedonic eating in men^39^. Although the exact mechanisms underlying the suppressive effects of OT on eating remain to be fully uncovered^27,28^, current understanding supports a combination of both metabolic and cognitive effects^40^, including modulation of reward-related signalling^41,42^. In addition, some small clinical studies starting to explore the potential of intranasal OT in improving disordered eating in patients with BN have shown that intranasal OT (40 IU) reduces caloric intake^43^ and decreases vigilance towards angry faces^44^ in women with BN. We have recently shown that a divided dose of intranasal OT (64 IU) modulates risk-taking behaviour in women with BN/BED^45^ and increases vigilance towards food, vs. neutral, images in a dot probe task in both women with and without BN/BED^46^. However, in the same cohort of women with BN/BED, we failed to find any effect of the same divided dose of intranasal OT (64 IU) on eating behaviour or stress response^47^. Apart from the lack of clarity regarding the effectiveness of intranasal OT to improve eating symptomatology in patients with BN/BED, it is also not clear which brain circuits intranasal OT targets in BN/BED patients.

In this study, we investigate abnormalities in resting brain physiology in women with BN/BED using ASL. Studying the brain at rest allows us to investigate baseline alterations in brain physiology that are not restrained to the specific neural circuits engaged by tasks. We further investigate whether an acute dose of 40IU of intranasal OT can restore alterations in resting brain physiology in BN/BED 18–26 min post dosing. We have previously shown that ASL captures OT-induced changes as early as 15–36 min post dosing in resting rCBF after a single acute intranasal administration (40 IU) in both healthy men^48,49^ and men at clinical high-risk for psychosis (CHR-P)^50^ (earlier time intervals have simply never been studied). We specifically address two questions: (1) Do women with BN/BED, compared with healthy women, present alterations in resting brain perfusion, as measured using ASL MRI? (2) Can 40 IU intranasal OT restore or attenuate these resting rCBF alterations in women with BN/BED 18–26 min post doing?

## Methods

### Participants

We recruited 25 women meeting the DSM-5 criteria for either BN (*n* = 20) or BED (*n* = 5) and 27 women with no current or prior eating disorder. We excluded three participants (two healthy controls and one BN patient) due to a large discrepancy in the time post dosing that we sampled rCBF in the OT and placebo visits (it exceeded the duration of one rCBF scan, i.e., >8 min). We further excluded two healthy women due to corruption of the data files. Our final sample included 23 healthy and 24 women with BN/BED. Ethical approval for the study was granted by the London—Camberwell St Giles Research Ethics Committee (Reference: 14/LO/2115). All participants provided written informed consent and participated voluntarily. See Supplementary Materials for further details regarding inclusion/exclusion criteria. Our previous work has demonstrated that *n* = 16 per group is sufficient to quantify OT-induced rCBF changes in men using a between-subjects^49^ or within-subjects design^48^.

### Study design and procedure

We employed a double-blind, placebo-controlled crossover design. Participants visited our centre on three occasions: one screening and two experimental visits. During screening, we measured height, weight, and collected basic demographics. Participants then completed the Eating Disorder Examination—Questionnaire (EDEQ) Version^51^ and Depression, Anxiety, and Stress Scales^52^ online in their own time between the screening and the first experiment visits, to minimize burden during the visits. Both questionnaires have been validated for online application, with online responses matching well the responses of participants in the controlled environment of the lab^53,54^. To habituate all participants to the scanner environment and minimize its potential distressing impact, all participants were first trained in a mock-scanner.

The experimental visits were conducted 2 days apart, ensuring participants were tested in the same phase of the oestrous cycle for both treatment conditions (OT and placebo). Each participant was asked to report the first day of their last menstrual period and any hormonal contraception they were currently taking. Participants were asked to eat 2.5 h prior to each experimental visit, to control for baseline hunger. All participants were tested at approximately the same time in the early evening (5–7 pm) for both the OT and placebo treatments, to minimize potential circadian variability in resting brain activity^55^ or OT levels^56^. Fifty minutes after arrival, participants self-administered 40 IU intranasal OT (Syntocinon, 40 IU/ml; Novartis, Basel, Switzerland) in ten puffs, one puff every 30 s, each puff containing 0.1 ml Syntocinon (4 IU) or placebo (same excipients as Syntocinon except for OT) and alternating between nostrils, over a period of 5 min. Participants were randomly allocated to a treatment order (OT/placebo or placebo/OT). After drug administration, participants were guided to the MRI scanner, where a single pulsed continuous ASL scan (8:20 min) was acquired 18–26 min (±4 mins) post dosing. Our choice of OT dose and post dosing interval was driven by our previous work, whereby we have shown that 40 IU intranasal OT induce robust changes in rCBF in both healthy men (as early as 15–32 min post dosing)^48,49^ and men at CHR-P (at 22–36 min)^50^. Furthermore, previous clinical studies have shown beneficial effects of a dose of 40 IU intranasal OT in women with BN^43,44^.

### MRI data acquisition

We used a three-dimensional (3D) pseudo-continuous ASL (3D-pCASL) sequence to measure changes in rCBF over 18–26 min post dosing. Participants were instructed to lie still, maintain their gaze on a centrally placed fixation cross during scanning and to let their mind flow (as per current practice for the acquisition of open-eyes resting-state data^57^). Labelling of arterial blood was achieved with a 1825 ms train of Hanning-shaped RF pulses in the presence of a net magnetic field gradient along the flow direction (the *z*-axis of the magnet). After a post-labelling delay of 2025 ms, a whole-brain volume was read using a 3D interleaved “stack-of-spirals” Fast Spin Echo readout^58^, consisting of 8 interleaved spiral arms in the in-plane direction, with 512 points per spiral interleave. Echo time (TE) was 11.088 ms and repetition time (TR) was 5135 ms. Fifty-six slice partitions of 3 mm thickness were defined in the 3D readout. The in-plane field of view (FOV) was 240 × 240 mm. The spiral sampling of *k*-space was re-gridded to a rectangular matrix with an approximate in-plane resolution of 3.6 mm. Each sequence used five control-label (C-L) pairs. Individual CBF maps were computed for each of the perfusion weighted difference images derived from every C-L pair, by scaling the difference images against a proton density image acquired at the end of the sequence, using identical readout parameters. This computation was done according to the formula suggested in the recent ASL consensus article^59^. The sequence uses four background suppression pulses to minimize static tissue signal at the time of image acquisition. We acquired eight 3D-pCASL sequences, with the duration of the entire acquisition time of each sequence being 8:20 min.

A 3D high-spatial-resolution, magnetization prepared rapid acquisition T1-weighted scan was also acquired (FOV of 270 mm, TR/TE/TI = 7.328/3.024/400 ms). The final resolution of the T1-weighted image was 1.1 × 1.1 × 1.2 mm.

### MRI data pre-processing

A multi-step approach was performed for the spatial normalization of the CBF maps to Montreal Neurological Institute (MNI) space: (1) co-registration of the proton density image from each sequence to the participant’s T1 image after resetting the origin of both images to the anterior commissure. The transformation matrix of this co-registration step was then applied to the CBF map, to transform the CBF map to the space of the T1-image; (2) unified segmentation of the T1 image; (3) elimination of extra-cerebral signal from the CBF map, by multiplication of the “brain only” binary mask obtained in step (2), with each co-registered CBF map; (4) normalization of the subject’s T1 image and the skull-stripped CBF maps to the MNI152 space using the normalization parameters obtained in step (2). Finally, we spatially smoothed each normalized CBF map using an 8 mm Gaussian smoothing kernel. All of these steps were implemented using the ASAP (Automatic Software for ASL processing) toolbox (version 2.0)^60^. The resulting smoothed CBF maps were then entered into Statistical Parametric Mapping (SPM) 12 (http://www.fil.ion.ucl.ac.uk/spm/software/spm12/) for group-level statistical analysis, as described below.

### Statistical analyses

#### Diagnosis, treatment and diagnosis × treatment effects on resting rCBF

To investigate the effects of diagnosis and treatment on global CBF, we first extracted mean global CBF values with an explicit binary mask for grey matter using the fslmeants command (FSL suite, http://www.fmrib.ox.a.c.uk/fsl). The binary mask was derived from a standard T1-based probabilistic map of grey-matter distribution by thresholding all voxels with a probability > 0.20. We tested for the main effects of treatment or diagnosis and for the treatment × diagnosis interaction on global grey-matter CBF signal using mixed analysis of variance and the Greenhouse-Geisser correction against violations of sphericity.

We then tested the effects of diagnosis, treatment and diagnosis × treatment on mean rCBF values extracted using *fslmeants* from 14 regions-of-interest (ROIs) corresponding to anatomical areas shown to be affected in BN/BED in previous structural, functional or neurochemical studies^7,8^. A detailed description of these ROIs can be found in Supplementary Fig. S1. The VTA, SN and hypothalamus masks were retrieved from a recently published high-resolution probabilistic atlas of human subcortical brain nuclei^61^. The OFC mask included the areas 14 and 11 m, and the medial PFC included area FPm from the connectivity-based parcellation map in Franz-Xaver Neubert et al.^62^. All the remaining masks were retrieved from the Harvard-Oxford Atlas distributed with FSL. To create the dorsal striatum masks, we pooled together in one single mask the caudate and putamen masks from the Harvard-Oxford Atlas. For the insula, amygdala, accumbens and dorsal striatum, we decided to consider right and left homologous structures separately, as we have previously described some degree of left lateralization of the effects of intranasal OT on rCBF in men^49,63^. We used a full factorial linear mixed model, including diagnosis, treatment and diagnosis × treatment as fixed effects, participants as a random effect and global grey-matter CBF as a nuisance variable. All analyses were implemented in SPSS24 (http://www-01.ibm.com/software/uk/analytics/spss/). Results are reported at a level of significance *α* = 0.05. For the ROI analyses, we contained the false discovery rate for the number of ROIs tested at *α* = 0.05 using the Benjamini–Hochberg procedure^64^.

Finally, we conducted a whole-brain exploratory investigation of treatment, diagnosis and treatment × diagnosis effects on rCBF, using global grey-matter CBF as a covariate (see Supplementary Materials for details). We used cluster-level inference at *α* = 0.05 using family-wise error correction for multiple comparisons and a cluster-forming threshold of *P* = 0.005 (uncorrected). These statistical thresholds had been determined a priori based on our own work investigating the effects of intranasal OT on rCBF in humans^48,49^ and are standardly applied in ASL studies measuring rCBF^65–70^.

#### Associations between clinical measures and rCBF in women with BN/BED and healthy controls

To investigate whether changes in rCBF in women with BD/BED were related to the severity of clinical symptomatology, we correlated the mean rCBF in each of the four anatomical ROIs where we found significant differences between the BN/BED and healthy groups with clinical measures. We focused our analyses on mean rCBF values extracted from anatomical ROIs to avoid potential issues of selection bias that might have emerged if we had based our analyses on rCBF extracted from clusters showing significant diagnostic group differences in the whole-brain analyses. As a measure of eating symptom severity, we used the global EDEQ scores. As patients with BN/BED score highly on scales measuring anxiety, stress and depression, and scores on these scales were highly correlated with each other, we used within-group principal component analysis (PCA) on these three measures to obtain a single score reflecting affective/stress symptom severity. The first principal component accounted for 70.81% and 83.47% of the total variance in the patients and healthy controls, respectively, and was used in our analyses to examine the specificity of the association of rCBF with eating symptom severity.

We estimated partial Pearson’s correlation coefficients (with bootstrapping—1000 samples) between mean rCBF and global EDEQ or the first component of the stress, anxiety and depression scores in each ROI, adjusting for global CBF and body mass index (BMI). To examine the specificity of the association between mean rCBF and eating symptomatology, we re-estimated the partial correlations with global EDEQ after including scores on the first principal component from the PCA on the stress, anxiety and depression measures. We estimated these correlations separately in patients and healthy controls, because group differences in mean scores on these measures might result in illusory correlations if the two samples were pooled together^71^. For completeness, we then compared these correlations between the patient and healthy control groups using the Fisher *r*-to-*z* transformation.

### Post-hoc analyses

#### BMI

As BMI influences perfusion in the brain^72^ and patients presented high variability in BMI, we repeated all our analyses including BMI as a nuisance variable to account for BMI-related variability in rCBF.

#### Hormonal contraception

Hormonal contraception can reduce intranasal OT-induced effects on brain physiology, at least in response to social stimuli^73^. As 37.5% of our women were under hormonal contraception, we repeated all analyses including hormonal contraception as a nuisance variable.

#### Diagnostic category, psychiatric comorbidities and current pharmacological treatment

To disentangle whether the main effect of diagnosis was driven specifically by BED status, current pharmacological treatment or the presence of other psychiatric conditions, we repeated our analyses for the main effect of diagnosis either excluding the five BED patients or including diagnostic category and comorbidities/current treatment as nuisance variables.

#### Grey-matter volume

Variations in grey-matter volume (GMV) can be associated with local variations in brain tissue metabolic demand, thereby influencing rCBF^74,75^. For this reason, we explored whether the main effects of diagnosis were related to differences in GMV. For each participant, we used GM volume probability maps obtained after segmentation of the T1-weighted structural image and the *fslmeants* command to estimate mean GMV values in ROIs based on the clusters where BN/BED patients showed significant increases in rCBF compared with controls in the whole-brain analyses (details provided in Supplementary Materials). We then repeated the whole-brain analysis testing for the main effects of diagnosis on rCBF including GMV as a covariate. For completeness, we also ran an exploratory whole-brain analysis testing for the main effect of diagnosis on GMV (further details in Supplementary Material). Our main motivation was to investigate if clusters showing significant effects of diagnosis in rCBF mapped onto brain areas showing significant GMV abnormalities, giving us insight in the interpretation of rCBF abnormalities in BN/BED.

## Results

### Sample characteristics

Women with BN/BED and healthy controls did not differ in age, height, BMI, educational level, or hormonal status (see Table 1). Women with BN/BED had significantly higher weight, anxiety, depression and stress scores, and EDEQ global and subscale scores compared with healthy women. Women with BN/BED had an average duration of eating disorder of 10.15 years and the average age of onset was 15.90 years old. Of the 25 women with BN/BED, 7 women had a comorbid psychiatric disorder. Specifically, five women had comorbid depression, four women had comorbid generalized anxiety disorder, four women had borderline personality disorder, one woman had social anxiety, one woman had obsessive-compulsive disorder, and one woman had an autism spectrum disorder. At the time of the study, seven women were taking an antidepressant, one woman was taking a mood stabilizer, and one woman was taking an antipsychotic drug. Participants with BN/BED reported an average binge-eating frequency of 14.14 episodes over the past 28 days (SD = 9.88). The women with BN endorsed an average frequency of self-induced vomiting equal to 10.40 occasions over the past 28 days (SD = 13.61), an average laxative abuse frequency of 5.13 occasions over the past 28 days (SD = 8.35), an average frequency of “hard exercise intended to control weight or shape” equal to 7.31 occasions over the past 28 days (SD = 8.57), and 1 participant reported using diuretic pills on 4 occasions over the past 28 days.

**Table 1:** Sociodemographic characteristics.

Variable	Healthy controls	BN/BED	Statistic	*p*
Number	23	25 (20 BN; 5 BED)	-	-
Age (years)	23.6 (3.79)	25.58 (6.31)	T(42) = 1.231	0.225
Height (cm)	162.85 (5.95)	166.38 (7.64)	T(46) = 1.684	0.099
Weight (kg)	59.19 (5.51)	67.13 (15.22)	T(46) = 2.160	0.037*
BMI (kg/cm^2^)	22.57 (1.89)	24.19 (5.21)	T(46) = 1.407	0.166
RQF (education)	5.31 (1.67)	4.875 (1.75)	T(42) = 0.836	0.408
Hormonal status				
Contraception	41.7%	32.0%		
Follicular phase	29.2%	28.0%	*χ*^2^ (3) = 5.090	0.165
Luteal phase	8.3%	32.0%		
Non-available	20.8%	8%		
Duration ED	-	10.15 (5.80)	-	-
Age onset ED	-	15.90 (5.07)	-	-
Comorbidities	0%	28%	-	-
Medication use	0%	20%	-	-
Frequency (last 28 days):				
Binge eating		14.14 (9.88)		
Self-induced vomiting		10.40 (13.61)		
Laxative abuse	-	5.13 (8.35)	-	-
Excessive exercising to control shape		7.31 (8.57)		
Eating disorder questionnaire (EQED)				
Global EQED	1.17 (1.12)	3.91 (1.30)	T(43) = 7.794	<0.0001*
Restraint	0.91 (0.90)	3.05 (1.76)	T(43) = 5.240	<0.0001*
Eating concern	0.64 (0.86)	3.51 (1.39)	T(43) = 8.521	<0.0001*
Weight concern	1.35 (1.52)	4.32 (1.40)	T(43) = 7.048	<0.0001*
Shape concern	1.77 (1.57)	4.76 (1.39)	T(43) = 6.994	<0.0001*
Depression score	3.05 (4.49)	20.39 (12.31)	Mann–Whitney *U* = 55	<0.0001*
Anxiety score	1.16 (2.52)	11.83 (9.56)	Mann–Whitney *U* = 37	<0.0001*
Stress score	3.68 (5.04)	20.33 (11.69)	Mann–Whitney *U* = 14	<0.0001*

This table shows the sociodemographic characteristics of our cohort of healthy and BN/BED women. Data are presented as mean (SD). The fourth and fifth columns summarize the statistics corresponding to groups comparison for each variable, when applicable. Statistical significance was set to *p* < 0.05 and is highlighted with the symbol *.

### Drug blinding

Participants guessed the drug condition correctly on 57 out of the total 104 visits (54.8% of visits), which was not significantly different from chance (one-sample binomial test, *z* (103) = 57.00, *p* = 0.377).

### Diagnosis, treatment and diagnosis × treatment effects on resting global CBF

There were no effects of diagnosis, treatment or diagnosis × treatment on global grey-matter CBF (Supplementary Fig. S2).

### Diagnosis, treatment and diagnosis × treatment effects on resting rCBF

#### ROI analyses

**Fig. 1:**
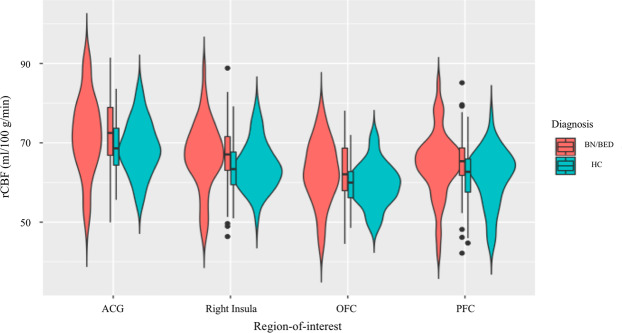
Increases in mean resting regional cerebral blood flow (rCBF) in the anterior cingulate, right insula, orbitofrontal and medial prefrontal cortices in BN/BED women compared with healthy women (hypothesis-driven analysis). These graphs illustrate the changes in mean rCBF in BN/BED patients compared with healthy controls for the regions-of-interest where we identified a significant main effect of diagnosis. For all of these four regions-of-interest, BN/BED women presented higher mean rCBF than healthy women. ACG anterior cingulate gyrus, OFC orbitofrontal cortex, PFC medial prefrontal cortex. Box plots and violin plots depicting mean rCBF (marginal means) on each region-of-interest for each diagnosis/treatment groups; middle horizontal lines represent the median; boxes indicate the 25th and 75th percentiles.

Women with BN/BED showed significant rCBF increases in the medial PFC and OFC ROIs compared with healthy controls (Supplementary Table S1). These differences did not survive correction for the number of total ROIs tested. Accounting for BMI, women with BN/BED showed significant rCBF increases in the medial PFC, OFC, right insula and ACG gyrus ROIs (Table 2 and Fig. 1), which remained significant after correcting for multiple testing.

**Table 2:** Effects of diagnosis, treatment and diagnosis × treatment on resting regional cerebral blood flow (rCBF) within neural circuits relevant for BN/BED (hypothesis-driven analysis).

Region-of-interest	Main effect of treatment			Main effect of diagnosis			Interaction treatment × diagnosis		
	*F*	*P* (uncorrected)	*P* (adjusted)	*F*	*P* (uncorrected)	*P* (adjusted)	*F*	*P* (uncorrected)	*P* (adjusted)
VTA	0.727	0.396	1.386	0.296	0.588	0.915	0.003	0.960	0.960
SN	1.400	0.240	1.680	0.027	0.871	1.016	0.006	0.939	1.095
Right Amy	0.154	0.696	1.624	4.231	0.043	0.120	0.008	0.927	1.179
Left amy	0.122	0.727	1.272	3.71×10^-4^	0.985	0.985	0.282	0.596	1.192
PFC	1.979	0.163	2.282	7.462	0.008	0.028	0.107	0.744	1.302
Orbitofrontal	0.105	0.746	1.160	9.100	0.003	0.042	0.005	0.944	1.016
Insula right	0.058	0.811	1.032	7.854	0.006	0.028	0.970	0.327	1,526
Insula left	0.038	0.846	0.846	1.264	0.264	0.528	0.038	0.846	1.184
HPT	1.315	0.254	1.185	0.254	0.616	0.862	0.076	0.784	1.219
ACG	0.134	0.715	1.430	8.347	0.005	0.035	0.596	0.442	1.031
Dorsal striatum right	0.080	0.778	1.089	2.983	0.088	0.205	0.965	0.329	1.152
Dorsal striatum left	0.054	0.817	0.953	0.019	0.892	0.961	0.985	0.324	2.268
Acc right	0.304	0.583	1.632	0.657	0.420	0.735	0.802	0.373	1.044
Acc left	0.040	0.842	0.907	0.042	0.838	1.067	1.200	0.276	3.864

This table shows the results of a hypothesis-driven investigation of the effects of diagnosis, treatment and diagnosis × treatment on rCBF within 14 anatomical regions-of-interest suggested to be involved in BN/BED. We tested these effects in a liner mixed model, controlling for global grey-matter cerebral blood flow and BMI. Statistical significance was set to *p* < 0.05, after correction for multiple testing with the Benjamini–Hochberg procedure.

*Acc* accumbens, *ACG* anterior cingulate gyrus, *Amy* amygdala, *PFC* prefrontal cortext, *HPT* hypothalamus, *SN* substantia nigra, *VTA* ventral tegmental area.

#### Whole-brain analysis

**Fig. 2:**
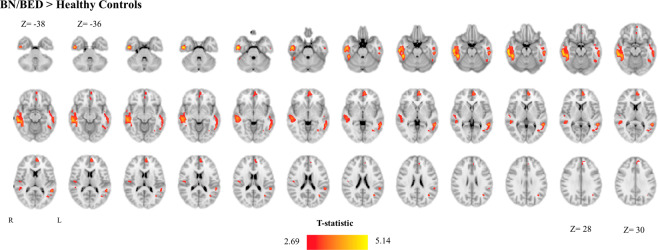
Increases in resting regional cerebral blood flow (rCBF) in the brain of BN/BED patients (whole-brain analysis). This figure shows the results of a directed T-contrast analysis at the whole-brain level where we tested for increases (BN/BED > Controls) or decreases (Controls > BN/BED) in rCBF in patients compared with controls, accounting for global grey-matter cerebral blood flow and BMI. Whole-brain cluster-level inference was applied at *α* = 0.05 using family-wise error (FWE) correction for multiple comparisons and a cluster-forming threshold of *p* = 0.005 (uncorrected). Images are shown as T-statistic in radiological convention. We did not find any significant cluster BN/BED patients presented lower rCBF than healthy controls.

Exploratory whole-brain analysis revealed rCBF increases in BN/BED patients in three clusters (Supplementary Fig. S3 and Supplementary Table S2). Two clusters spanned the inferior/middle temporal gyrus bilaterally (with a bigger extent at the right lobe) and one cluster the PFC, medial OFC and ACG bilaterally (extending more into the left hemisphere). Accounting for BMI extended the right inferior/middle temporal gyrus cluster to include the right posterior insula (Fig. 2 and Supplementary Table S3).

We did not observe any treatment or treatment × diagnosis effects.

### Associations between clinical symptoms and rCBF in women with BN/BED and healthy controls

**Fig. 3:**
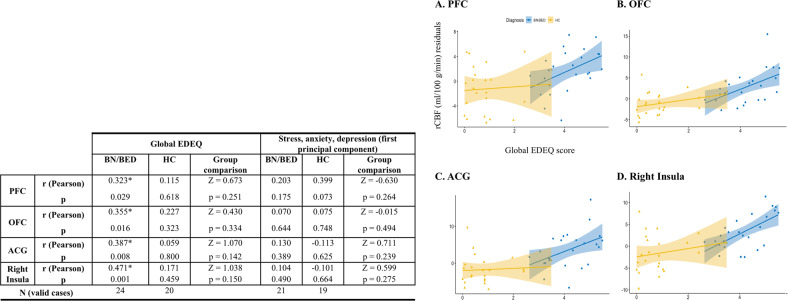
Increases in resting regional cerebral blood flow (rCBF) in the brain of BN/BED patients correlate positively with eating disorder symptom severity. Left panel: partial Pearson correlations between mean rCBF in each of the four anatomical regions-of-interest where we found significant differences between the BN/BED and healthy groups, and clinical symptomatology. We used the global EDEQ scores as a measure of eating disorder symptom severity and the first principal component of the anxiety, stress and depression scores. Partial Pearson correlations were calculated with bootstrapping (1000 samples) adjusting for global CBF and BMI, separately for controls and patients. In the last columns of each sub-section, we present the result of the statistical comparison of the correlations between the two groups, as assessed by Fisher’s *r*-to-*z* transformation. Right panel, we present scatter plots depicting the relationship between mean rCBF (marginal scores after regressing out the effects of global CBF and BMI in the *y*-axis) and global EDEQ scores (in the *x*-axis) in each region-of-interest. Statistical significance was set to *p* < 0.05 and is highlighted with the symbol *. PFC medial prefrontal cortex, OFC orbitofrontal cortex; ACG anterior cingulate gyrus.

We observed significant positive partial correlations (adjusting for BMI and global CBF) between mean rCBF extracted from the medial PFC, OFC, ACG and right insula ROIs and global EDEQ scores, in patients but not in controls (Fig. 3). Partial correlations between mean rCBF in these ROIs and global EDEQ were still significant after additionally accounting for stress, anxiety and depression (Supplementary Table S4), except for the medial PFC ROI for which the correlation was at trend-level (*p* = 0.051). However, direct comparisons of these correlations between the two groups yielded no statistically significant differences (Fig. 3 and Supplementary Table S4).

### Post-hoc analyses

#### Hormonal contraception

Accounting for use of hormonal contraception produced no change in our findings (Supplementary Table S5 and Supplementary Fig. S4).

#### Diagnostic category, psychiatric comorbidities and current pharmacological treatment

Excluding the BED patients (Supplementary Fig. S5) or accounting for diagnostic category (Supplementary Fig. S6) as a covariate in our model produced did not substantially change our results. We also did not find any substantial changes in our results when we accounted for comorbidities/current drug treatment (Supplementary Fig. S7).

### Is regional GMV associated with the effect of diagnosis in rCBF?

The main effect of diagnosis in the whole-brain analysis remained virtually unaltered after accounting for GMV values in the ROIs based on the clusters where BN/BED patients showed significant increases in rCBF compared with controls in the whole-brain analyses (Supplementary Fig. S8). Furthermore, women with BN/BED showed lower GMV than controls in two clusters, one spanning the postcentral and precentral gyri and another the inferior/middle temporal gyrus, both in the right hemisphere (Supplementary Fig. S9). Interestingly, the right temporal gyrus cluster partially overlapped with the cluster showing rCBF increases in women with BD/BED compared with controls (Supplementary Fig. S9). In the right temporal gyrus, rCBF and GMV correlated negatively in patients but not in controls (Supplementary Fig. S10) (BN/BED: *r* = −0.496, *p* = 0.012; healthy controls: *r* = −0.024, *p* = 0.913; Groups comparison: *z* = −1.66, *p* = 0.048). We did not identify any area in the brain where patients presented higher GMV than controls.

## Discussion

Using ASL, a highly reproducible and widely available quantitative MRI technique, we demonstrated, for the first time, resting hyperperfusion abnormalities in women with BN/BED, compared with healthy controls, in key neural circuits implicated in BN/BED psychopathology. Hyperperfusion abnormalities in the PFC, medial OFC, ACG and the right posterior insula were positively and specifically associated with symptom severity as reflected on the global EDEQ scale in women with BN/BED. However, a single dose of 40 IU intranasal OT did not restore or attenuate these perfusion abnormalities at 18–26 min post dosing. Our findings enhance our understanding of resting brain abnormalities in BN/BED and identify resting rCBF as a non-invasive potential biomarker for disease-related changes and treatment monitoring. We discuss each of our main findings below in turn.

### Abnormalities in resting brain perfusion in women with BN/BED compared with healthy controls

Our hypothesis-driven analyses showed that women with BN/BED, compared with healthy controls, presented with increased resting rCBF in the medial PFC, medial OFC, ACG and the right posterior insula. Exploratory whole-brain analysis showed further increases in the inferior/middle/superior temporal cortices in women with BN/BED. Sensitivity analyses confirmed that these findings were not driven by the inclusion of BED patients and did not reflect current medication or other psychiatric comorbidities. In women with BN/BED, estimates of rCBF in three key areas where we found women with BN/BED and healthy women to differ in rCBF (medial PFC, ACG and right insula) correlated positively with global scores on the EDEQ, even after adjusting for anxiety, depression and stress, supporting a specific association between rCBF abnormalities in these regions and the underlying eating disorder psychopathology. Given the high reliability and ease of acquisition, our findings suggest that measuring resting rCBF at BN/BED patients with ASL MRI can offer a non-invasive in-vivo biomarker of potential value for understanding disease-related functional changes, posing minimal demand and no risk on patients. In addition, future large-scale studies should characterize the sensitivity and specificity of changes in resting rCBF in the identified brain regions in terms of distinguishing between patients and controls, or distinguishing between patients who respond to specific treatments from those who do not.

We are aware of only three studies examining rCBF in BN/BED, using SPECT and small sample sizes (7 < *n* < 14). One study found increased rCBF in the right temporal lobe in response to own body images when a pooled sample of patients with DSM-IV BN-purging type and anorexia nervosa-restrictive type were compared with a pooled sample of healthy controls, patients with DSM-IV BN-non-purging type and patients with anorexia nervosa-purging type^17^. Another study reported increased rCBF in the left temporal lobe and inferior frontal region bilaterally in patients with DSM-III BN before eating^16^. Finally, the only study examining patients with BED reported that obese BED patients, compared with non-obese BED patients and healthy controls, presented increased rCBF responses in the frontal and prefrontal cortices in response to food visual stimuli^18^. These studies did not report on rCBF changes at rest, which precludes direct comparisons between these studies and ours. Nevertheless, we note that the changes in rCBF reported in these studies are in the same direction and largely overlap with regions where we found resting hyperperfusion during BN/BED in our study. Therefore, it is possible that hyperperfusion in these areas in women with BN/BED marks a disease-relevant state, which can be captured at rest and without the need of a specific experimental manipulation.

The mechanisms behind these increases in resting rCBF in BN/BED remain unknown. Increases in resting rCBF are likely to reflect basal regional hypermetabolism, potentially associated with increases in resting neural activity^15^. We note however that the few small-scale studies evaluating resting brain glucose metabolism using PET during BN have generally reported decreases in global and regional glucose metabolism, including parietal and anterior frontal hypometabolism^76–80^. The only exception is one study that found increased glucose metabolism in the temporal lobes of patients with BN, which matches our findings of increased perfusion in these areas^78^. BN patients have been reported to present higher GMV in some areas such as the medial OFC^8^, which could account for the increases in rCBF we report herein; however, we found that this is unlikely to be the case in our sample. Exploratory whole-brain analysis showed decreased GMV in the right temporal lobe in BN/BED compared with controls. This cluster partially overlapped with one cluster showing increased rCBF in patients. GMV was negatively correlated with rCBF in this area in patients, but not in controls. We speculate that two possible mechanisms could account for this paradoxical relationship between rCBF and GMV in the temporal lobe of patients with BN/BED. One possibility is that the increases in rCBF we observed in the right temporal lobe might reflect a mechanism of local functional plasticity in response to GMV loss in this region, which ultimately may help to maintain temporal lobe function in patients with BN/BED^81,82^. Alternatively, it is also conceivable that enduring neural hyperactivity, accompanied by increases in rCBF, could drive GMV loss due to excitotoxicity^83^. Future longitudinal studies examining how changes in GMV and rCBF in these brain regions develop may elucidate these hypotheses.

As our data were acquired at rest, we can only speculate regarding the specific contribution of the perfusion and structural abnormalities observed in this study to core behavioural manifestations in BN/BED. We note that BN/BED patients have been reported to present with enhanced sensitivity to the anticipation of food reward/hedonic value^5^, which matches the increases in rCBF we observed in the medial OFC (a key area involved in reward/outcome value processing^84^, including the hedonic value of food^85^). BN, in particular, and, to some extent, BED have also been associated with continuous feelings of monitoring of binge-activating stimuli in the environment and body shape/weight self-judgement, which match our findings of increased rCBF in the medial PFC and ACC (two key areas involved in self-monitoring and control regulation^86^) in patients. Evidence from lesion studies have supported a direct implication of the temporal lobe (predominantly the right lobe) in eating disorders^87^. Although the exact mechanisms by which temporal lobe dysfunction may contribute to eating dysregulation remain elusive, some studies have implicated the superior/middle temporal gyri in the brain responses to palatable food^88^ and in inhibitory control, including cognitive control of appetite^89^—two core elements of binge eating. Importantly, previous studies have reported attenuated recruitment of these areas during response inhibition in both BN^90,91^ and BED^92^.

In contrast to our hypotheses, we could not find any resting rCBF alterations in the ventral or dorsal striatum in patients with BN/BED. This is surprising, given the pivotal role of the striatal circuits in cognitive processes known to be disrupted in BN/BED (such as incentive and habitual behaviours^93^, impulsivity^94^ and self-regulation^95^) and previous neuroimaging studies demonstrating functional and structural abnormalities in these areas (see refs. ^7,8^ for detailed reviews). It is possible that while disrupted rCBF in the striatum in BN/BED may not manifest at rest, alterations may become evident in condition engaging this region, such as the anticipation or valuation of hedonic stimuli (e.g., food).

### Does intranasal OT restore rCBF abnormalities in women with BN/BED?

The lack of treatment or treatment × diagnosis effects does not support a normalizing effect of 40 IU intranasal OT on resting rCBF abnormalities in women with BN/BED 18–26 min post dosing. A number of reasons might explain the lack of effects of intranasal OT on rCBF in this study. First, it is possible that we may have missed the active time-window for intranasal OT treatment effects in women with BN/BED. We have previously mapped intranasal OT-induced effects on brain perfusion in men and demonstrated that while 40 IU intranasal OT can induce changes in resting perfusion as early as 15 min post dosing (earlier intervals have not been sampled), the effects do vary as a function of method of administration, dose and the latency of the sampling interval post dosing^48^. However, we are still lacking an in-depth pharmacodynamics investigation of OT-induced changes in rCBF in women. Previous studies have shown that the effects of the administration of the same dose of intranasal OT to men and women can result in different effects on behaviour and brain function^96–100^. A recent study has shown that differences in the effects of a range of doses of intranasal OT on brain responses to happy and fearful faces between men and women do not simply reflect differences in dose sensitivity, but rather gender-specific effects^101^. Therefore, although we have informed this current study based on our in-depth characterizations of the pharmacodynamics of intranasal OT in men, it is possible that crucial differences exist in the spatiotemporal pattern of rCBF changes after intranasal OT between genders. Future studies should systematically investigate the potential effects of these factors, including a comparison of acute vs. chronic regimens of administration, characterization of rCBF changes over an extended period of time and dose–response studies in women. Second, hormonal contraception has been shown to blunt responses to intranasal OT in women^73^. Although accounting for contraception in our analyses produced virtually no change in our results, it is plausible that OT might have exerted an effect on rCBF in our sample in the absence of hormonal contraception. Finally, while we could not detect any significant treatment effects on rCBF in women with or without BN/BED at rest in this study, we cannot exclude that significant effects of intranasal OT might emerge during targeted experimental challenges, such as exposure to food cues^102^ or stress^103^. Indeed, we have recently shown that a divided dose of intranasal OT (64 IU) modulates risk-taking behaviour in women with BN/BED^45^ and increases vigilance towards food, vs. neutral, images in a dot probe task in both women with and without BN/BED^46^ (but does not affect eating behaviour or stress response^47^), using the same sample.

### Limitations

One limitation of our study is that we only included women. Our findings should thus not be extrapolated to men with BN/BED. Second, we could only enrol a small number of BED patients, which did not allow us to isolate effects related to diagnostic category. Third, although we have asked subjects to eat 2.5 h before our experimental sessions in order to minimize the effects of baseline hunger, we did not standardized the amount or type of food consumed, which may have introduced some noise in our data. Lastly, 20% of our BN/BED sample had other psychiatric comorbidities and 28% were under current pharmacological treatment, respectively, which may have confounded our results. We have investigated the impact of such factors in a sensitivity analysis where we repeated our main analyses including current treatment and comorbidities as a nuisance variable. Although our findings remained largely unaltered, future replication in samples of women with BN/BED and without these confounds would be beneficial.

## Conclusions

BN/BED in women is accompanied by increased resting perfusion in key brain areas potentially associated with the underlying eating psychopathology. Intranasal OT did not attenuate or restore these abnormalities, at least for the specific dose and post-dosing interval examined. Future studies examining a more comprehensive range of doses, time-windows and schemes of administration will be needed before we can ascertain the therapeutic value of intranasal OT in improving resting rCBF disturbances in women with BN/BED. Given the high reliability and ease of acquisition, measuring resting rCBF at BN/BED patients with ASL MRI offers a promising non-invasive in-vivo biomarker of functional changes in these patients, with potential implications for diagnosis and treatment monitoring.

## Supplementary information

Supplementary Information

## Data Availability

Data can be provided upon request.
